# First record of predation of *Nyctinomops laticaudatus* (É. Geoffroy, 1805) by *Chrotopterus auritus* (Peters, 1856) (Mammalia: Chiroptera)

**DOI:** 10.3897/BDJ.7.e38303

**Published:** 2019-08-28

**Authors:** Ianna Sonegheti Borloti, Vinícius Teixeira Pimenta, Albert David Ditchfield

**Affiliations:** 1 Centro de Investigação em Biodiversidade e Recursos Genéticos da Universidade do Porto (CIBIO-UP), Departamento de Biologia, Faculdade de Ciências da Universidade do Porto, Porto, Portugal Centro de Investigação em Biodiversidade e Recursos Genéticos da Universidade do Porto (CIBIO-UP), Departamento de Biologia, Faculdade de Ciências da Universidade do Porto Porto Portugal; 2 Centro de Ciências Humanas e Naturais, Departamento de Ciências Biológicas, Universidade Federal do Espírito Santo - UFES, Vitória, Brazil Centro de Ciências Humanas e Naturais, Departamento de Ciências Biológicas, Universidade Federal do Espírito Santo - UFES Vitória Brazil

**Keywords:** Atlantic Forest, bat, behavior, carnivory, diet, Phyllostominae, Vampyrini

## Abstract

The Wooly False Vampire Bat, *Chrotopterus auritus* (Peters, 1856) (Chiroptera: Phyllostomidae), feeds on small mammals, birds, lizards, frogs and occasionally large insects and fruits. In this paper we report an additional evidence of bat predation by *C. auritus*. A male of this species was captured with a partially eaten Broad-eared Free-tailed Bat, *Nyctinomops laticaudatus* (É. Geoffroy, 1805) (Chiroptera: Molossidae). This record was obtained during a research project conducted in the Biological Reserve of Sooretama, Southeastern Brazil.

## Introduction

Neotropical bats have the highest diversity of dietary habits among mammals, including nectar, fruits, blood, insects and vertebrates (Kunz et al. 2011). Several Phyllostominae (Phyllostomidae) species occasionally feed on small vertebrates, but the prevalence of regular carnivory is unique to the tribe Vampyrini (Baker et al. 1989; Bonato et al. 2004; Giannini and Kalko 2005; Simmons 2005). This correlation between phylogeny and feeding habits reinforces the importance of dietary studies within Phyllostomidae.

*Chrotopterus auritus* is a large Vampyrini that feeds on small mammals, birds, frogs, reptiles, insects, and occasionally fruits (Peracchi and Albuquerque 1976; Medellin 1988; Giannini and Kalko 2005). In Phyllostomidae and Noctilionidae, the consumption of small vertebrates is associated with increased body size (Peracchi and Albuquerque 1976; Wetterer et al. 2000). The species *C. auritus* may present opportunistic feeding habits (Wolda 1988; Bonato et al. 2004). However, according to Bonato et al. (2004), small mammals represent more than 70% of the biomass consumed by that species, and may include bats. Nevertheless, cases of consumption of bats by *C. auritus* are very rare (Nogueira et al. 2006).

Acosta y Lara (1951) reported predation on *Glossophaga soricina* (Pallas, 1766); Bordignon 2005 reported the predation of *Carollia perspicillata* (Linnaeus, 1758) and *Peropterix macrotis* (Wagner, 1843) in a cave. Nogueira et al. 2006 also reported predation of *C. perspicillata*, which was found partially eaten in a mist net. Brito et al. 2010 caught *C. auritus* with a half-eaten *Tadarida brasilienses* (I. Geoffroy, 1824). Witt and Fabián 2010 identified *Myotis sp*. through fecal pellet analyses. Predation of the species *Nyctinomops laticaudatus* had never been reported so far. This paper increases the number of known bat species preyed on by *C. auritus*.

## Methods

This research was conducted in the Biological Reserve of Sooretama, a protected area of 24,250 hectares formed by a large area of primary Atlantic Forest, located mainly in the municipality of Sooretama but also spanning to Jaguaré, Linhares and Vila Valério in northern Espírito Santo, Southeastern Brazil. The climate is Tropical (Am in Koppën's classification), with a rainy season in the summer and a short dry season in the winter. Average temperature is 23 ºC, and annual rainfall is 1250.5 mm. The dominant vegetation is lowland dense tropical rainforest (MMA 2007).

**Figure 1.**
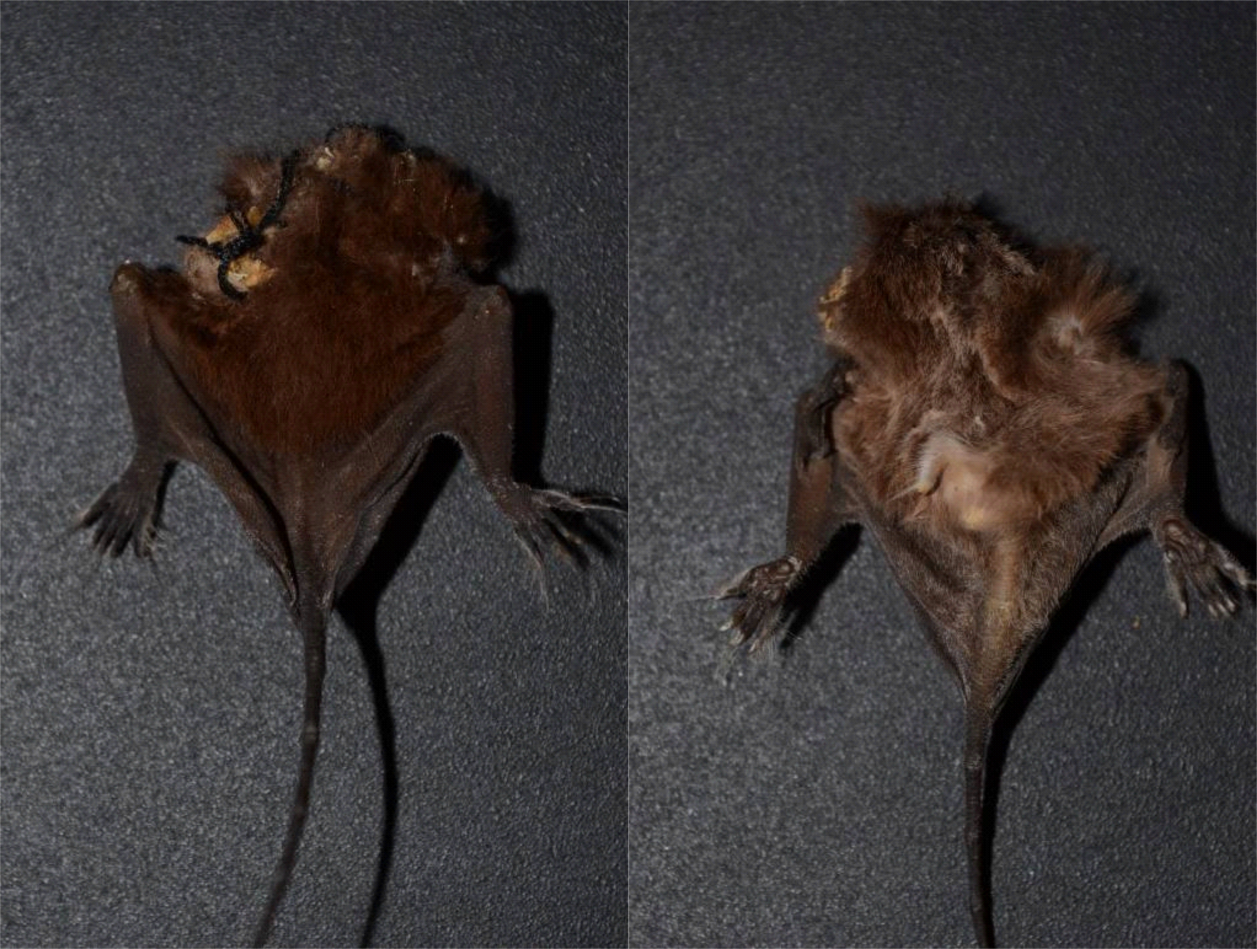
Dorsal (left) and ventral (right) views of a *Nyctinomops laticaudatus* preyed by *Chrotopterus auritus*.

Capture of the bats was performed using mist nets placed at ground level, on a trail in the far eastern region of the conservation unit (19°02'44"S and 39°57'30"W, elevation of 38 m). An adult male of *C. auritus* was captured in December 2011. Simultaneously, 15 cm below it, the remains of a molossid bat, the lower body intact, were recovered (Fig. 1). The voucher specimen of *C. auritus* was placed in 70% ethonol for long-term preservation and deposited at "Laboratório de Estudo em Quirópteros" (LABEQ), Universidade Federal do Espírito Santo (UFES), Brazil, under the code VP450. The individual of *C. auritus* had feces and intestinal contents collected and its components were analyzed using a stereoscopic microscope. The fragments that could represent parts of bats were separated to assist in identifying the prey.

## Results

**Figure 2.**
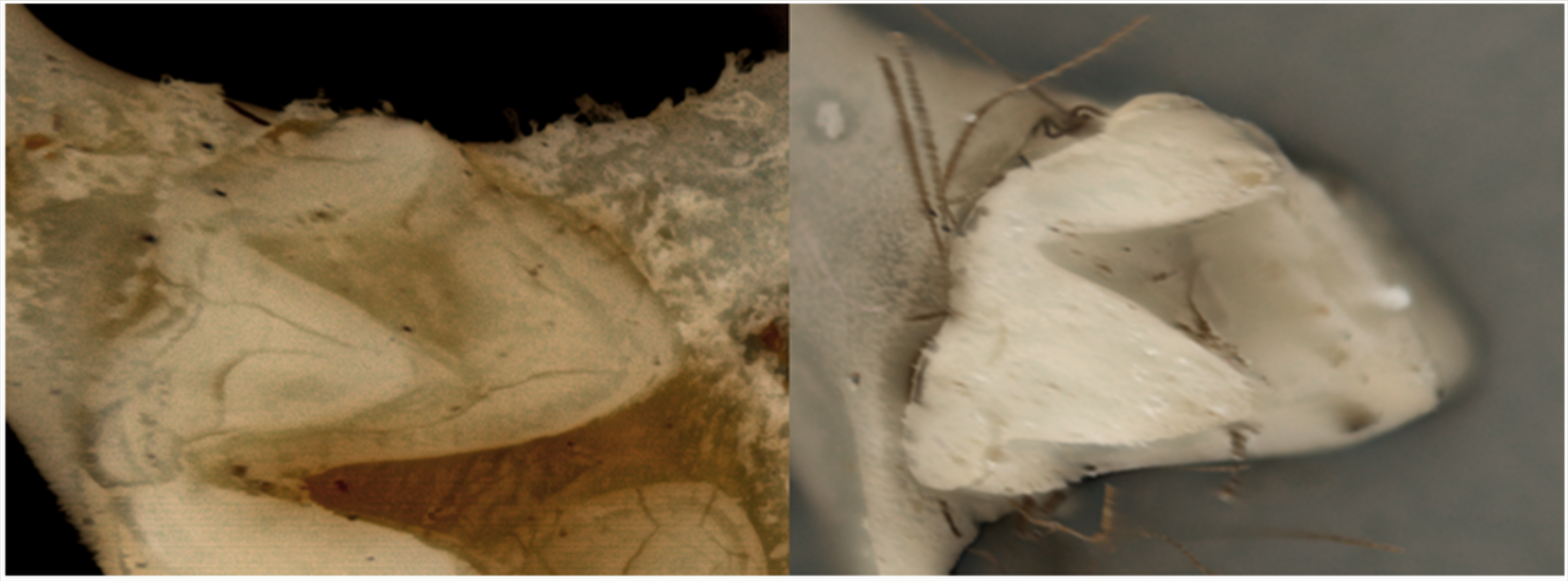
P3 premolar of *Nyctimomops laticaudatus*. On the left a specimen with skull intact; on the right the tooth found among the intestinal contents of *Chrotopterus auritus*.

In the intestinal contents, an intact tooth was found, and we could identify it as a premolar (P3) of *Nyctinomops laticaudatus* (Fig. 2) using identification keys (Gregorin and Taddei 2002; Gregorin and Cirranello 2015). The specimen has a dark brown dorsal coat, with lighter brown hair with whitish tips on the ventral region. The hair around the toes are almost twice as long as the toes themselves (Fig. 1). Measurements of external structures were taken for comparison with other species of this family occurring in the region. A specimen of *Nyctinimops macrotis*, found in the same locality by Hoppe et al. (2014), was included in this comparative analysis (Table 1). No fragments of mist net were found in the intestinal tract, feces, or even in the mouth of the specimen of *C. auritus*, and the net around the body of the molossid bat was intact.

## Discussion

This work adds a new species of bat to the list of preys of *C. auritus* (see Acosta y Lara 1951; Bordignon 2005; Nogueira et al. 2006; Brito et al. 2010), being the second species in the family Molossidae recorded in the wild under natural conditions. In captivity, Peracchi and Albuquerque 1976 recorded consumption of a *Molossus* sp. after a few days of starvation.

*Nyctinomops laticaudatus* had not been recorded as prey of another bat species. This record demonstrates versatility in the diet of *C. auritus*. Bats of the family Molossidae have long narrow wings in proportion to body size, which makes them fast-flying animals with limited maneuverability (Avila-Flores et al. 2002). Because of these characteristics they are considered best adapted for the role of foragers in open spaces, in a continuous hunting flight. On the other hand, the load-carrying capacity is very important for carnivorous bats taking large prey. Their shorter and broader wings would enhance maneuverability and the ability to hunt in cluttered surroundings (Norberg and Fenton 1988). This type of wing structure would be more energetically expensive for continuous flight. Because of that, *Chrotopterus auritus* is more a sit-and-wait predator than a fast hunter. In fact, some authors have referred to *C. auritus* as an opportunistic feeder (e.g., Sazima and Sazima 1978; Bonato et al. 2004).

It is important to emphasize that this record does not provide information about the time and place where predation occurred. Thus, there are two possible scenarios: 1) the predation of *N. laticaudatus* occurred while this individual was in the mist net; 2) the predation occurred elsewhere and therefore *C. auritus* was captured with its prey. Complementary studies are needed in order to know more details regarding the diet and feeding behavior of *C. auritus*.

## Figures and Tables

**Table 1.** Comparative Morphometrics of the specimen that suffered predation (VP 450p) with other Molossidae in the area. Legend: TL – tail length; LTL – left tibia length; RTL – right tibia length; LFL – left foot length; RFL – right foot length; FLF – greater length of fur of left foot; FRF – greater length of fur of right foot. *Animal caught in the municipality of Vitória/ES, Brazil. All others from the Biological Reserve of Sooretama.

**Species**	**TL**	**LTL**	**RTL**	**LFL**	**RFL**	**FLF**	**FRF**
***Nyctinomops laticaudatus***	44,8	14,8	14,7	9,1	9,1	7,0	7,1
***Nyctinomops laticaudatus***	42,1-46,9	14,1-15,0	14,1-14,9	9,0-9,2	9,1-9,5	7,0-7,1	7,0-7,2
Nyctinomops macrotis*	57,6	20,3	20,1	10,6	11,1	7,3	7,5
***Molossus coibensis***	35,6-37,1	14,0-14,1	13,9-14,2	8,4-9,0	8,8-9,0	4,3-5,3	4,6-5,2
***Molossus molossus***	36,2-38,9	14,1-15,4	14,1-15,3	8,9-10,0	9,2-9,7	4,1-5,0	3,8-5,2
***Molossus rufus***	49,0-52,5	20,6-21,3	21,3-22,7	11,1-12,1	11,1-12,0	4,6-5,8	4,7-5,4
***Eumops glaucinus***	51,6-52,5	22,2-24,0	22,2-24,8	11,9-12,0	12,2-12,3	5,0-5,1	5,1-5,6
